# Diagnosis of aphasia in stroke populations: A systematic review of language tests

**DOI:** 10.1371/journal.pone.0194143

**Published:** 2018-03-22

**Authors:** Alexia Rohde, Linda Worrall, Erin Godecke, Robyn O’Halloran, Anna Farrell, Margaret Massey

**Affiliations:** 1 School of Health and Rehabilitation Sciences, The University of Queensland, Brisbane, Queensland, Australia; 2 School of Medical and Health Sciences, Edith Cowan University, Joondalup, Western Australia, Australia; 3 Department of Community and Clinical Allied Health, La Trobe University, Melbourne, Victoria, Australia; 4 Department of Speech Pathology, Royal Brisbane and Women's Hospital, Brisbane, Queensland, Australia; Centro de Neurociencias de Cuba, CUBA

## Abstract

**Background and purpose:**

Accurate aphasia diagnosis is important in stroke care. A wide range of language tests are available and include informal assessments, tests developed by healthcare institutions and commercially published tests available for purchase in pre-packaged kits. The psychometrics of these tests are often reported online or within the purchased test manuals, not the peer-reviewed literature, therefore the diagnostic capabilities of these measures have not been systematically evaluated. This review aimed to identify both commercial and non-commercial language tests and tests used in stroke care and to examine the diagnostic capabilities of all identified measures in diagnosing aphasia in stroke populations.

**Methods:**

Language tests were identified through a systematic search of 161 publisher databases, professional and resource websites and language tests reported to be used in stroke care. Two independent reviewers evaluated test manuals or associated resources for cohort or cross-sectional studies reporting the tests’ diagnostic capabilities (sensitivity, specificity, likelihood ratios or diagnostic odds ratios) in differentiating aphasic and non-aphasic stroke populations.

**Results:**

Fifty-six tests met the study eligibility criteria. Six “non-specialist” brief screening tests reported sensitivity and specificity information, however none of these measures reported to meet the specific diagnostic needs of speech pathologists. The 50 remaining measures either did not report validity data (n = 7); did not compare patient test performance with a comparison group (n = 17); included non-stroke participants within their samples (n = 23) or did not compare stroke patient performance against a language reference standard (n = 3). Diagnostic sensitivity analysis was completed for six speech pathology measures (WAB, PICA, CADL-2, ASHA-FACS, Adult FAVRES and EFA-4), however all studies compared aphasic performance with that of non-stroke healthy controls and were consequently excluded from the review.

**Conclusions:**

No speech pathology test was found which reported diagnostic data for identifying aphasia in stroke populations. A diagnostically validated post-stroke aphasia test is needed.

## Introduction

Aphasia affects up to 42% of stroke survivors [1] and impacts on a person’s verbal expression, auditory comprehension, reading and/or writing [2]. Post-stroke language intervention has been found to assist in optimising patient outcomes [3], consequently accurate aphasia diagnosis is crucial in ensuring patients receive the rehabilitation they require [4,5].

The accuracy of aphasia diagnostic procedures has important implications in stroke care. Epidemiological studies vary significantly with respect to their diagnostic criteria for aphasia [6] often leading to variations in incidence and prevalence statistics [7]. Stroke studies estimate that anywhere between 15% [8] to 42% [1,9] of acute stroke patients experience language impairment. The global burden of stroke is high; in 2013 the prevalence of stroke was 25.7 million, with 10.3 million people experiencing a first-time stroke [10]. With an incidence of 10.3 million new strokes internationally, these differing epidemiological statistics have significant ramifications on a global level and result in differences in estimated affected global populations anywhere between 1.5 and 4 million annually. The accurate, methodologically sound diagnostic validation of post-stroke aphasia assessments is consequently pivotal in ensuring appropriate funding and provision of healthcare resources [6] and is an important component of global stroke healthcare.

Post-stroke language functioning is currently evaluated through a range of clinical measures and assessments in acute clinical care. Neuroimaging studies have identified high correlations between lesion site and aphasia, where site and size of lesion have been found to be important factors in predicting recovery [11,12]. While these imaging methods contribute to understanding loss of language functions by characterizing the lesion [13], they do not report on the nature and individual profile of language impairment which is dependent instead upon bedside testing and clinical assessment of language functioning [14].

A wide range of language tests are currently used in post-stroke care [15]. Stroke scales such as the European Stroke Scale (ESS) [16], Canadian Neurological Scale (CNS) [17] and National Institutes of Health Stroke Scale (NIHSS) [18] gauge acute stroke severity and include subtest items which evaluate acute language functioning. These measures are used to inform hyperacute stroke treatment decision making and while they are often used to identify stroke patients with aphasia, they have not been specifically validated for this purpose [4] and do not assist with diagnostically differentiating between aphasic and non-aphasic stroke populations.

Brief screening tests such as the Frenchay Aphasia Screening Test [19] and Language Screening Test [20] have been specifically designed to assess post-stroke language performance. These tests are designed for general use by multiple ‘non-specialist’ health professionals [4,21,22] to identify at-risk patients and ensure prompt referral [4, 19–23]. Such language assessments typically assess a narrow range of language abilities [24], frequently omitting reading/writing tasks [20,24] and consequently are not considered suitable for use in isolation for diagnostic purposes [7].

Speech pathologists are typically responsible for diagnosis of aphasia resulting from stroke [25]. Tests used to assist in this clinical decision making usually evaluate a range of language skills, identify communicative strengths and weaknesses, aid in planning treatment and assist with a definitive diagnosis of language impairment [26]. Speech pathologists often have only a brief window, frequently around 30 minutes, in which to conduct a thorough clinical examination of acute language functioning [14]. Vogel et al [15] found that within acute hospital settings, stroke patients typically undergo initial speech pathology language assessment within 2 days of admission. Despite the often highly variable patient performances during this acute recovery phase, logistical demands dictate that clinicians need to make swift diagnostic decisions or run the risk of patients being missed and lost post discharge [27]. Despite the likelihood that some patients’ difficulties may resolve [14], accurate aphasia diagnosis ensures that appropriate follow-up procedures are implemented.

El Hachioui et al [4] conducted a systematic review which aimed to identify and examine the diagnostic validation of post-stroke language screening tests. Validation studies for eight screening tests [19–24,28,29] were identified which reported the tests’ ability to differentiate between aphasic and non-aphasic stroke populations. Despite this review’s systematic evaluation of the published research literature, the authors stated that no research study was found which aimed to diagnostically validate a stroke language assessment that took longer than 15 minutes to administer. While brief screening measures for ‘non-specialist’ clinicians have diagnostic validation studies published in the peer-reviewed research literature, there is a lack of similar published psychometric information for longer, more comprehensive stroke language measures for speech pathologists.

El Hachioui et al [4] and others [15,30] have commented on the notable absence of published diagnostic validation for commonly used speech pathology tests such as the Western Aphasia Battery-Bedside [31], Acute Aphasia Screening Protocol [32] and Aachen Aphasia Bedside Test [33]. Vogel et al [15] noted that their search of research databases failed to produce any articles on the validity of the most commonly used speech pathology test, the Mount Wilga High Level Language Test [34]. Similarly, in their systematic review Salter et al [30] noted the absence of research literature evaluating the measurement properties of other commonly used tests such as the Bedside Evaluation Screening Test (BEST-2) [35], Sklar Aphasia Scale [36], Aphasia Screening Test [37] and Aphasia Language Performance Scales [38]. While these tests are frequently used in stroke care [15], these longer, more comprehensive language assessments often report their psychometrics within their purchased test manuals or through online sources and not within peer-reviewed journals. As a consequence, the diagnostic capabilities of these language tests have not been systematically evaluated.

Given the importance of prompt, accurate identification of acute post-stroke language deficits in stroke management, this review had two main aims. Firstly, to identify both commercially published and other non-commercially available adult language tests. Tests were identified through two sources; firstly, through a systematic search of commercial and academic publishers, stroke and speech pathology resource webpages and other professional websites; secondly, from language tests which have been reported to be used by clinicians in stroke care. The second aim of the review was to examine the test manuals, materials or any associated psychometric resources of all identified tests to determine which language tests compared patient test performance against that of a reference standard language measure and reported sensitivity and specificity data in differentiating between aphasic and non-aphasic stroke populations. This review aims to comply with the PRISMA guidelines for systematic reviews [39] (S1 Table).

## Methods

### Test identification and search strategy

Tests were collected from a systematic search of the following academic and commercial publishing websites, speech pathology and stroke websites and resource webpages and professional websites: ABC-CLIO, AbeBooks, Academic Press, Academic Press Corporation, Adam Matthew Digital, Alexander Street Press, Allen Press, Allied Publishers, Amazon, American Book Company (1996), American Psychological Association, Ann Arbour Publishers, Aphasia Institute, Arena (Australian publishing co-operative), ASHA (Store), Ashgate Publishing, Bedford-St. Martin's (“Macmillan Learning”), Bentham Science Publishers, Bepress, Berg Publishers, Berghahn Books, BioMed Central, BioOne, Bioscientifica, BookDepository, Booktopica, Boydell & Brewer, Brill Publishers, Brunswick Books, BUROS: Centre For Testing, Caister Academic Press, Cambria Press, Carl Hanser Verlag, Carl Heymanns Verlag, Carolina Academic Press, CCD Publishing, Channel View Publications, Chemistry Central, CNKI, Cold Spring Harbor Laboratory Press, College Publications, Co-op, Copernicus Publications, Dunedin Academic Press, DVV Media Group, Dymocks, E. Schweizerbart, EBSCO Information Services, EDP Sciences, Edward Elgar Publishing, ELife Sciences Publications, Elsevier, Emerald Group Publishing, Flat World Knowledge, Freund Publishing House, Future Medicine, Global Speech Therapy Direct, Gorgias Press, Gotland Museum, Greenbranch Publishing, Hayden-McNeil, Henry Holt and Company, Hindawi Publishing Corporation, Hogrefe, Humana Press, Inderscience Publishers, Informa, Ingenta, InteLex Past Masters, International Medical Press, International Universities Press, IOP Publishing, IOS Press, Ivyspring International Publisher, JMIR Publications, John Benjamins Publishing Company, John Donald (imprint), Jones & Bartlett Learning, Karger Publishers, Königshausen & Neumann, Landes Bioscience, Legenda (imprint), Libertas Academica, Linguisystems, Lippincott Williams & Wilkins, Litwin 43 Books, LLC, Living Reviews, M. E. Sharpe, Maney Publishing, Martinus Nijhoff Publishers, Mary Ann Liebert, Inc., MDPI, The Medical Letter, Inc., Medknow Publications, Mettler & Salz, Mohr Siebeck, NASW Press, Nature Publishing Group, Nauka (publisher), Naukova Dumka, Neura, Nova Science Publishers, OMICS Publishing Group, Open Court Publishing Company, Ovid Technologies, Palgrave Macmillan, Papery Open Science Aggregated, PAR Inc., Pearson Clinical, Peerage of Science, PeerJ, Peeters (publishing company), Pensoft Publishers, Perspectivia.net, Peter Lang (publisher), Pickering & Chatto Publishers, Pluto Press, Polity (publisher), Pro-Ed (Australia), Pro-Ed Inc., Pulsus Group, Rodopi (publisher), Routledge, Rowman & Littlefield, SAGE Publications, Verlag Anton Saurwein, Sciences Nat, Scientific Research Publishing, Sinauer Associates, Smithsonian Institution Press, Springer, Springer Nature, Springer Publishing, Springer Science + Business Media, SPW Publishing, Stroke Engine, T&T Clark, Taylor & Francis, Technika (publisher), Technosphera (publisher), Telos (journal), The Nile, The Therapy Store, Thieme Medical Publishers, Trove National Library of Australia, Tsehai Publishers, Ubiquity Press, Ukrainian Encyclopedia (publishing), Universal Publishers (United States), University of Hertfordshire Press, University of Minnesota Press, University Press of America, Urban & Schwarzenberg, Wageningen Academic Publishers, Wharton School Publishing, Wiley-Blackwell, Winslow Resources, Wolters Kluwer, Woodhead Publishing, Wordery, World Scientific.

The following search strategy was applied for Booktopia ‘Popular Medicine & Health’ [keyword search], ‘aphasia’, ‘Language & Linguistics’ [keyword search], ‘Psychology’ [keyword search], ‘aphasia test’, ‘language test’, ‘dysphasia.’ Further search terms included: condition (aphasia OR dysphasia OR language) setting (acute OR bedside OR hospital OR poststroke) population (stroke OR CVA OR brain OR intracran* OR ischemia OR intracranial OR thrombosis OR hemorrhage test) and instrument (screen OR tool OR assessment OR instrument OR evaluation OR protocol OR inventory OR index OR profile). The search strategy was adapted for each website, resource page or publisher’s database. No further search limits or restrictions on publication date were applied. The reference lists of the selected tests were checked to detect additional publications. The search was completed 5 May 2017.

To ensure findings replicate clinical practice and to identify tests in use but no longer in publication, language tests reported to be used by speech pathologists in their stroke care [15] were also included. Vogel et al [15] sent an email survey to 254 practicing speech pathologists providing stroke care asking clinicians to report their language assessment practices in acute (<30 days post) stroke. Email lists were obtained through clinical directories of national professional bodies and special interest groups. Sixty eight percent (174) of the speech pathologists completed the questionnaire all of whom identified aphasia as part of their clinical caseload. Tests reported by clinicians in this survey request were included within the analysis.

### Test selection

Documents were eligible for inclusion if they were designed to assess language or language-based communication (e.g. functional language) in adults as defined as: impaired language functioning occurring anywhere across the severity spectrum from severe aphasia to mild conditions, occurring within any of the language modalities of verbal expression, auditory comprehension, reading, writing and gesture. Non-language specific tests (e.g. cognition), paediatric tests and documents not available in English were not included. Tests were excluded if they did not have psychometric resources or materials reporting on the test’s validity or if the study did not compare aphasic stroke test performance with that of a non-aphasic stroke comparison group. The aim of the study was to evaluate the ability of identified language tests to identify aphasia within stroke-only populations. Tests which used a study population of non-stroke aphasic patients such as those with disorders arising from traumatic brain injury, tumour or Alzheimer’s disease or for which the aetiology of aphasia for study participants was not stated were therefore excluded. Included studies were those able to report on the accuracy of the test in differentiating aphasic and non-aphasic stroke participants only, and not the test performance of the non-stroke aphasic patient. As per previous aphasia systematic reviews [4], this study aimed to identify tests suitable for use in stroke clinical practice. Tests with studies comparing the test performance of aphasic stroke patients with healthy controls (as opposed to stroke patients without aphasia) were also excluded.

Included tests were examined for documented psychometric ability to detect post-stroke aphasia. Tests had to report on results (sensitivity, specificity, likelihood ratios or diagnostic odds ratios) of patient test performance and that of a reference language measure. Studies could use cross-sectional or cohort design and had to report on (or enable calculation of) the sensitivity and specificity of the measure. Speech pathologists (or similar professions such as speech and language therapists, aphasiologists or neuropsychologists) were the target health professional. No test administration time limits were applied.

### Procedure and data extraction

All document titles obtained from the search were reviewed for eligibility. Where eligibility could not be determined from title alone, documents were retrieved and separately evaluated by two reviewers. Language tests meeting the initial criteria underwent validity analysis. Psychometric data was obtained directly from the language test manuals, published studies cited within manuals or from any associated online or published material or from published psychometric resources. Tests, test manuals and associated resources were obtained online, from The University of Queensland speech pathology departmental or library holdings or obtained via their international library loans system. Where manuals or associated resources were no longer in publication or in circulation and could not be sourced internationally via library networks, psychometric text books which directly reported on test manual’s psychometric data were consulted [26,40,41]. For each test, the presence and nature of validation studies were recorded. For tests reporting multiple similar psychometric studies, the study which most closely correlated with the study’s inclusion criteria was recorded. The type of study groups (target and/or comparison group) and aetiology/diagnosis of study participants were checked by two independent reviewers.

### Data analysis

Search results and test inclusion data were reported in a flowchart of document selection. Tests which underwent validity analysis were reported in a second flowchart of document selection and evaluation.

## Results

### Systematic search, test identification and validity analysis

**Fig 1:**
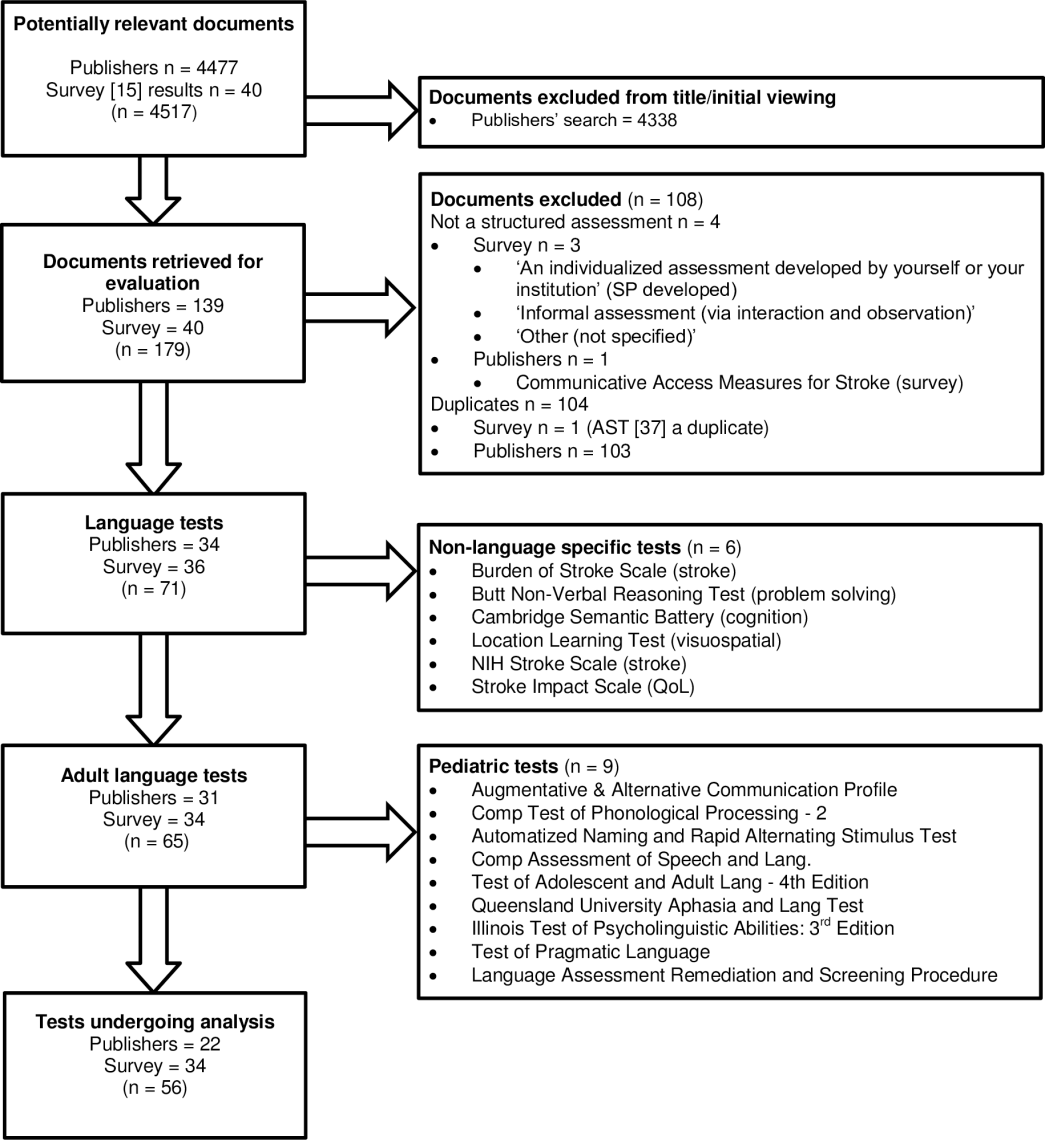
Flowchart of test selection.

**Fig 2:**
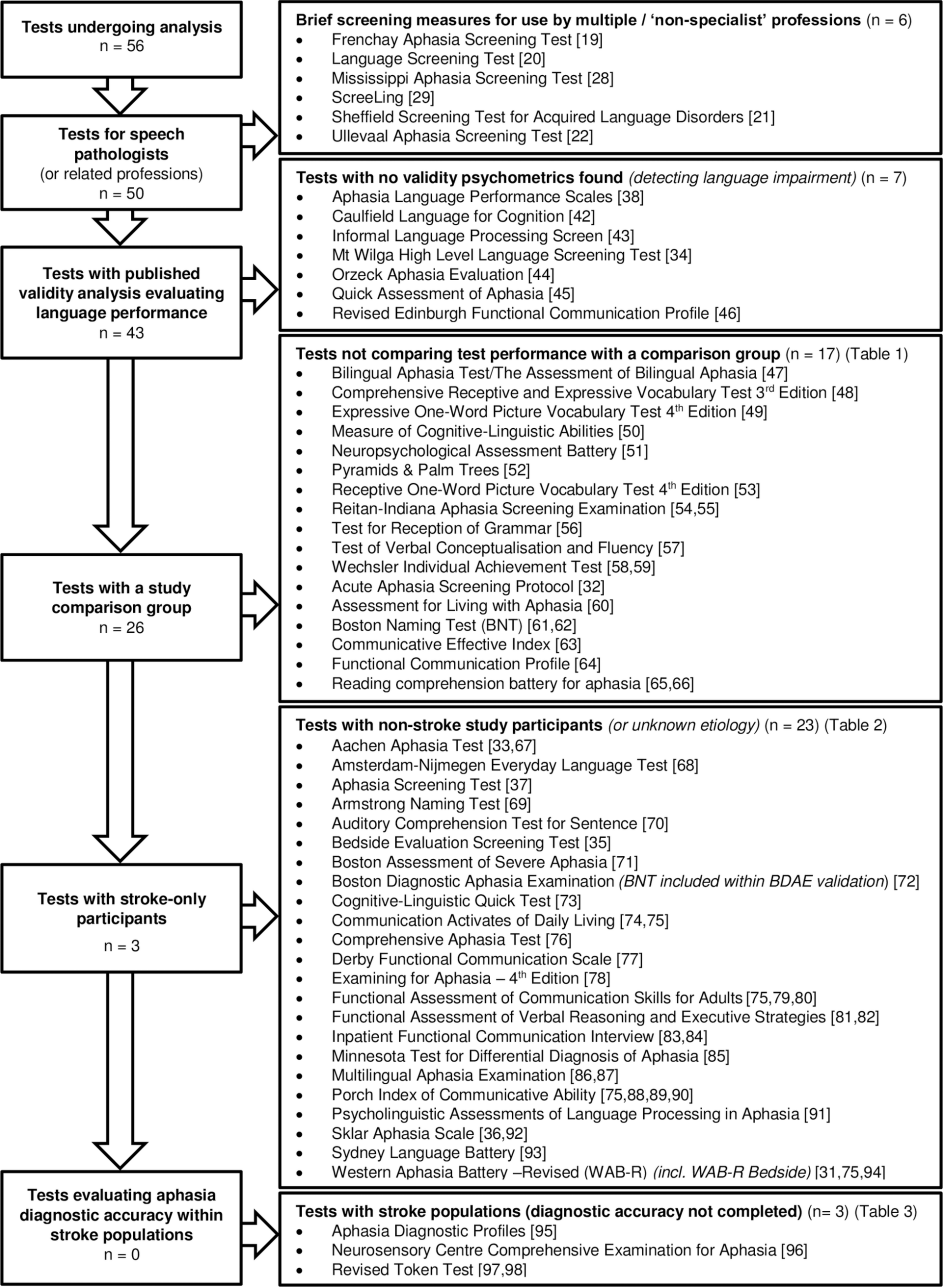
Flowchart of test exclusion based on validity analysis.

A total of 4517 documents were analysed. After screening titles, 139 documents from the database and online search and 40 tests from Vogel et al [15] were retrieved and evaluated (Fig 1). A total of 56 documents met the eligibility criteria and underwent psychometric validity analysis (Fig 2).

Tests and test studies did not meet the study eligibility criteria for a variety of reasons. Six tests were non-speech pathology specific measures intended for ‘non-specialist’ clinicians [19,20–22,28] or to assist with ‘adequate referral’ [29]. Of the 50 remaining tests, no validity data was found for seven measures [34,38,42–46]. Forty-three tests had published validity analysis, of these 17 had a single cohort consisting of either a non-language impaired (n = 11) [47–59] or an aphasic (n = 6) [32,60–66] participants group (Table 1). For these studies, no comparisons were made between the test scores of aphasic and non-aphasic study participants.

**Table 1:** Tests which did not compare test performance of language impaired with non-impaired language study participants (or language status not stated) (n = 17).

**Language test**	**Participant group: Non-impaired language (n = 11)**
**Bilingual Aphasia Test/The Assessment of Bilingual Aphasia** [47]	Multiple versions. Test development: 60 native speakers (non-brain-damaged, nonpsychotic patients) in the country where target language is spoken [47]
**Comprehensive Receptive and Expressive Vocabulary Test 3rd Edition (CREVT-3)** [48]	1,535 individuals demographically representative sample of the 2011 US population [48]
**Expressive One-Word Picture Vocabulary Test 4th Edition (EOWPVT)** [49]	2,400 individuals which resemble the US population [49]
**Measure of Cognitive-Linguistic Abilities (MCLA)** [50]	204 participants who lacked previous head injury or neurological involvement [50]
**Neuropsychological Assessment Battery** *(language component able to be administered in isolation)* [51]	1,448 adults that comprises two normative samples [51]
**Pyramids & Palm Trees** [52]	2 control groups of normal adults (no. not provided) [52]
**Receptive One-Word Picture Vocabulary Test 4**^**th**^ **Edition (ROWPVT)** [53]	(see [49]). Test was ‘co-normed’ with the EOWPVT-4 [49,53]
**Reitan-Indiana Aphasia Screening Examination** *(incorporated into Halstead-Reitan Neuropsychological Battery)* [54]	85 normal subjects [55]
**Test for Reception of Grammar (TROG-2)** [56]	70 healthy adults [56]
**Test of Verbal Conceptualization and Fluency (TVCF)** [57]	1,788 individuals between 8–89 years who approximate US demographics [57]
**Wechsler Individual Achievement Test** [58,59]	Adult normative data for ages 20–50 using nationally stratified sample [59]
**Language test**	**Participant group: Impaired language (n = 6)**
**Acute Aphasia Screening Protocol** [32]	48 acute aphasic patients [32]
**Assessment for Living with Aphasia (ALA)** [60]	101 patients with aphasia *(discrimination between severity levels but not aphasia vs*. *non-aphasia)* [60]
**Boston Naming Test (BNT)** *(BNT validation also included within BDAE validation)* [61,62]	100 aphasic patients [62]
**Communicative Effectiveness Index (CETI)** [63]	11 recent onset aphasia patients; 11 stable aphasic patients [63]
**Functional Communication Profile (FCP)** [64]	16 aphasic patients; 55 subjects with right hemiplegia associated with first stroke *(presence of language impairment inferred but not explicitly stated)* [64]
**Reading Comprehension Battery for Aphasia (RCBA)** [65]	26 aphasic adults [66]

Of the remaining 26 measures, 23 had studies which included non-stroke populations within their study samples [31,35–37,67–87,90–94] (Table 2). Non-stroke conditions included: head injured/ closed head injury/ trauma or traumatic brain injury [31,37,68,70,71,73,74,77,78,82,92,94], tumour or ‘neoplastic’ conditions [31,37,68,74,92,94], spinal cord injury [67], multiple sclerosis (MS) [67,77], Alzheimer’s disease [69,73], post-surgical conditions [70,84], hereditary spastic paraparesis [77], learning disability, learning disorder [78], chronic obstructive airway disease [84], hypoxic brain injury [84], schizophrenia [92], primary progressive aphasia [93], abscess, degenerative, aneurism [94], A-V malformation [94], Parkinson’s disease, Korsakoff’s syndrome [94] or aetiology not specified [31,68,71,82,85,92,94]. In addition, 19 of the 23 tests had studies [31,35,37,67–70,72–76,78,80,82,86,87,90–94] which evaluated the performance of aphasic participants against that of healthy controls. Two studies compared patient performance with either ‘communication intact’ [84] or ‘non-aphasic’ general medical ward patients [85], and two studies [71,77] did not include a non-aphasic comparison group. Three psychometric studies [75,78,82] were identified which examined test sensitivity and specificity. Ross & Wertz [75] evaluated the diagnostic accuracy of the Western Aphasia Battery (WAB) [31], Communication Activities of Daily Living (CADL-2) [74], American Speech-Language-Hearing Association’s Functional Assessment of Communication Skills for Adults (ASHA-FACS) [79] and Porch Index of Communicative Ability (PICA) [88]. LaPointe & Eisenson [78] evaluated the accuracy of the Examining for Aphasia Test 4^th^ Edition [78] and MacDonald & Johnson [82] evaluated the validity of the Adult FAVRES [81]. All three studies however included non-stroke aetiologies [78,82] and/or healthy controls [75,78,82] in their study samples and were excluded from the review.

**Table 2:** Tests with non-stroke populations included within study samples (or aetiology not specified) (n = 23).

Language test	Language-impaired group	Language intact group
**Aachen Aphasia Test (AAT)** [33]	120 aphasic patients [33]	
	135 adults with aphasia from stroke [67]	93 without aphasia (24 healthy controls, 41 hospitalised patients with no CNS damage; 28 with neurological illness (stroke, spinal cord injury or MS)) [67]
**Amsterdam-Nijmegen Everyday Language Test** [68]	260 aphasic patients (unilateral (left) lesion) and acquired aphasia of cerebrovascular aetiology (stroke 94%, trauma 3%, tumour 1%, other/unknown 2%) [68]	60 subjects (20–87 years) without history of neurological impairment or disease [68]
**Aphasia Screening Test** [37]	108 aphasics (stroke, tumour, head injury, haematoma, multi infarct) [37]	28 normal adult elderly [37]
**Armstrong Naming Test** [69]	20 aphasics; 20 probable Alzheimer’s disease [69]	25 normal elderly control [69]
**Auditory Comprehension Test for Sentences (ACTS)** [70]	150 aphasics (134 stroke; 10 TBI (traumatic brain injury); 6 post-surgical) [70]	30 normal controls [70]
**Bedside Evaluation Screening Test (BEST-2)** [35]	164 individuals with aphasia [35]	30 normal control subjects [35]
**Boston Assessment of Severe Aphasia (BASA)** [71]	111 aphasic patients (3 head injured; otherwise etiology not specified) [71]	
**Boston Diagnostic Aphasia Examination** [72]	85 aphasics [72]	15 elderly normal [72]
**Cognitive-Linguistic Quick Test (CLQT)** [73]	38 individuals with a history of neurological dysfunction (stroke, head injury, Alzheimer’s disease) [73]	170 nonclinical research sample [73]
**Communication Activities for Daily Living (CADL-2)** [74]	175 patients with neurologically based communication disorders (stroke, TBI, left-hemisphere cerebral tumours) [74]	30 participants without neurological damage [74]
	10 aphasia due to stroke [75]*	10 non-brain injured healthy controls [75]
**Comprehensive Aphasia Test (CAT)** [76]	266 subjects with aphasia [76]	27 non-aphasic normal controls [76]
**Derby Functional Communication Scale** [77]	16 hospital in-patients with acquired communication problems (stroke, TBI, MS, hereditary spastic parapesis) [77]	
**Examining for Aphasia Test - 4th Ed.** [78]	58 persons with aphasia (physical or health impairment, learning disability, learning disorder, deaf, TBI and other) [78]*	50 healthy elderly adults [78]
**Functional Assessment of Communication Skills for Adults (ASHA FACS)** [79]	15 post-stroke chronic aphasia [80]	15 healthy older people [80]
	10 aphasia due to stroke [75]*	10 non-brain injured healthy adults [75]
**Functional Assessment of Verbal Reasoning and Executive Strategies (Adult FAVRES)** [81]	52 acquired brain injury (46 trauma; 6 other) [82]*	101 individuals without brain injury [82]
**Inpatient Functional Communication Interview (IFCI)** [83]	9 hospital inpatients (hypoxic brain damage, stroke, chronic obstructive airway disease, and post-surgical *(test aims to assess general communication)* [84]	2 communication intact [84]
**Minnesota Test for Differential Diagnosis of Aphasia (MTDDA)** [85]	157 aphasic subjects [85]	50 non-aphasic normal medical ward patients [85]
**Multilingual Aphasia Examination** [86]	50 aphasic patients [86]	360 English speakers with nil history of hemispheric brain disease.; 61 healthy controls [86]
	48 aphasic subjects [87]	115 normal subjects [87]
**Porch Index of Communicative Ability (PICA)** [88,89]	150 aphasic left brain injured patients [88,89]	131 normal, non-brain injured adults [90]
	10 aphasia due to stroke [75]*	10 healthy non-brain injured adults [75]
**Psycholinguistic Assessments of Language Processing in Aphasia (PALPA)** [91]	25 subjects with aphasia post stroke [91]	32 non-brain-damaged subjects (generally partners of aphasics) [91]
**Sklar Aphasia Test** [36]	73 aphasics (vascular, traumatic, neoplastic and other) [92]	27 brain-damaged patients without aphasia (vascular, traumatic, neoplastic and other); 32 schizophrenics and 27 normal [92]
**Sydney Language Battery** [93]	57 patients (with Primary Progressive Aphasia) [93]	54 healthy controls [93]
**Western Aphasia Battery—Revised** *(including WAB-R Bedside)* [31]	10 aphasia due to stroke [75]*	10 healthy non-brain injured adults [75]
	150 aphasics (stroke, tumour, trauma, degenerative, aneurism, hemorrhage, A-V malformation, abscess and uncertain) [31,94]	59 non-aphasic controls (21 non-brain injured controls; 38 with various aetiologies: stroke, tumour, degenerative, hemorrhage, A-V malformation, Parkinson’s and Korsakoff’s) [31,94]
	215 aphasics (141 aphasics with infarcts (analysed separately), 74 aphasics due to other aetiologies: 34 tumour, 25 trauma and 15 miscellaneous) [31]	63 controls (10 normals and 53 non-aphasic patients with right hemisphere damage (aetiology not specified)) [31]

* sensitivity/specificity calculated

The final three tests (Table 3), the Aphasia Diagnostic Profiles (ADP) [95] the Neurosensory Centre Comprehensive Examination for Aphasia [96] and Revised Token Test [97] reported studies evaluating the performance of aphasic and non-aphasic stroke patients. While the ADP [95] examined patient test performance against stroke lesion site, neither this, nor the other studies [96,97,98] conducted diagnostic analysis comparing patient performance against a language reference standard. Of the 50 speech pathology tests included in the review, no study was located which reported diagnostic data differentiating between aphasic and non-aphasic stroke populations.

**Table 3:** Test studies that evaluated the performance of language-impaired and language-intact stroke patients (however, did not complete diagnostic accuracy (validity) analysis using a reference standard language measure) (n = 3).

Language test (n = 3)	Language-impaired group	Language-intact group
**Aphasia Diagnostic Profiles (ADP)** [95]	127 right-handed left hemisphere stroke patients *(non-language impaired left hemisphere stroke patients included within this sample)* [95]	39 bilateral stroke patients; 40 normal adults. *Patient ADP performance compared with presence or stroke lesion site as categorization criterion (not language diagnosis)* [95]
**Neurosensory Centre Comprehensive Examination for Aphasia (NCCEA) (Spreen-Benton Aphasia Tests)** [96]	206 aphasic patients [96]	Non-aphasic or normal group. *Patients scores compared against 3 different profiles*: *normal adults; aphasic and non-aphasic brain damaged patients (sensitivity/ specificity analysis between groups not completed)* [96]
**Revised Token Test** [97]	30 adults with aphasia [97]	
	30 left hemisphere with aphasia [98]	25 left hemisphere without aphasia; 53 right hemisphere *(sensitivity/ specificity analysis against reference standard not completed)* [98]

## Discussion

The aim of this review was twofold. Firstly, to identify adult language tests from a systematic search of commercial and academic publishers, stroke and speech pathology webpages and other professional websites and language tests reported to be used by clinicians in stroke care [15]. Secondly, to examine the test manuals, materials or any associated resources of all identified tests for reported psychometrics verifying the test’s ability to differentiate between aphasic and non-aphasic stroke populations. Fifty-six language tests were analyzed. Fifty of these tests were intended to meet the specific clinical needs of speech pathologists or similar professions. Of these measures, no study was located which reported diagnostic data in differentiating aphasic and non-aphasic stroke populations.

Some included tests appeared to indicate they had diagnostic capabilities (e.g. Boston Diagnostic Aphasia Examination [72] or Aphasia Diagnostic Profiles [95]). The Aphasia Quotient (AQ) from the WAB-R was designed to assist with distinguishing between aphasic and non-aphasic test performance however was psychometrically developed from test scores obtained from patients which included multiple different non-stroke aetiologies [31,94]. The WAB-R authors report that ‘the AQ alone cannot be used to label whether a brain damaged patient is aphasic’ [31: p.92]. Despite stroke being the leading cause of aphasia [99] none of these measures met the study’s inclusion criteria for diagnosing aphasia within stroke populations.

Overall, many tests included in the review either lacked validity data or did not include a comparison group within their study design. Where psychometric data was present, studies often analyzed a range of types of test validity (e.g. concurrent validity, construct validity) however test diagnostic accuracy was rarely examined, with only three studies [75,78,82] evaluating sensitivity and specificity. All three studies however included non-stroke participants in their study samples. The use of non-stroke participants was the single most significant factor for studies being excluded and accounted for 23 tests being eliminated from the review. In psychometric test validation, patient samples should be representative of the population in which the test is intended to be applied in practice [4,100]. In diagnosing post-stroke aphasia, aphasic stroke test performance needs to be compared against non-aphasic stroke patients [4]. Patients with language deficits resulting from non-stroke aetiologies (e.g. TBI, Parkinson’s disease, tumour) or the performance of healthy controls (who have not had a stroke) should not be included within either the aphasic or language-intact group as neither are representative of stroke patient populations [4,100]. The clinical application of these tests’ psychometrics in the identification of language deficits within stroke populations is therefore significantly limited. Finally, while three tests’ studies [95–98] examined aphasic and non-aphasic stroke patients’ performance, none evaluated the diagnostic accuracy of the test based on aphasia diagnosis.

The method of publication of language test studies may be a factor contributing to the quality of psychometric validation of the measures. The notable absence of peer-reviewed journal publications of the psychometrics for commonly used speech pathology tests has previously been reported [4,15,30]. While peer-reviewed published articles undergo significant appraisal prior to publication, commercially published measures or tests made available through other sources often lack an equivalent review process. In addition, the psychometrics of commercially published assessments are often available only upon purchase of the test. This publication method consequently can limit the systematic evaluation of the test’s diagnostic capabilities prior to purchase and use in clinical practice. The reporting of psychometric data in sources other than within the peer-reviewed journal literature may help account for why some studies, where present, did not adhere to traditional diagnostic test standards [101].

The findings of this review need to be considered in the context of the following study limitations. The access and location of test manuals and test psychometric data were a significant factor in conducting this review. Some tests listed online, on publication databases or those reportedly used in stroke care were no longer in circulation or available to access or purchase, and consequently these tests’ psychometrics were instead obtained through published normative text books. The reliability of the reporting on these psychometrics is therefore dependent upon the quality and comprehensiveness of the reporting within these texts and on some occasions this information was lacking. Where information was available, this was reported fully within the psychometric tables.

Tests in this review were also specifically examined for their psychometric capabilities in diagnosing acute post-stroke language deficits. It should be noted however that analysed tests had a variety of aims, including tests aimed at examining functional language abilities (e.g. Communication Activities of Daily Living (CADL-2) [74]) and assessments focused on assessing detailed elements of language functioning (e.g. PALPA) [91]). As such, not all tests were intended to be diagnostic instruments. Despite this, the review found that the psychometrics of many measures, irrespective of the tests’ aims were still often lacking with a number of measures not reporting analysis of test validity (of any form).

## Conclusion

Accurate diagnosis of aphasia is an important component of stroke care [5]. This review was unable to identify a speech pathology language test diagnostically validated for this purpose. Post-stroke aphasia diagnosis currently depends on a range of assessments, many of which lack validation, are inadequately standardised or intended for ‘non-specialist’ screening. The absence of research data informing this diagnostic decision-making does not adhere to evidence-based standards [102] and the accuracy of current diagnostic procedures is therefore unknown. Limited clinical skill or experience may lead to potentially missed or inaccurate diagnosis [15] and compromised stroke patient care. There is a need for a diagnostically robust speech pathology test for the identification of aphasia in stroke populations.

## Supporting information

S1 Table PRISMA checklist for systematic reviews. (DOCX)
